# Synthesis and Biological Evaluation of Novel 8-Morpholinoimidazo[1,2-*a*]pyrazine Derivatives Bearing Phenylpyridine/Phenylpyrimidine-Carboxamides

**DOI:** 10.3390/molecules22020310

**Published:** 2017-02-17

**Authors:** Shan Xu, Chengyu Sun, Chen Chen, Pengwu Zheng, Yong Zhou, Hongying Zhou, Wufu Zhu

**Affiliations:** 1 School of Pharmacy, Jiangxi Science & Technology Normal University, Nanchang 330013, China; Shanxu9891@126.com (S.X.); chen5950677@163.com (C.C.); Zhengpw@126.com (P.Z.); 2 Department of Pharmacy, The Affiliated Hospital of Chongqing Three Gorges Medical College, Chongqing 404000, China; zhouyong_0801@126.com (Y.Z.); 15223632072@163.com (H.Z.)

**Keywords:** imidazopyrazine, phenylpyridine-carboxamide, phenylpyrimidine-carboxamide, PI3Kα, synthesis

## Abstract

Herein we designed and synthesized three series of novel 8-morpholinoimidazo[1,2-*a*]pyrazine derivatives bearing phenylpyridine/phenylpyrimidine-carboxamides (compounds **12a**–**g**, **13a**–**g** and **14a**–**g**). All the compounds were evaluated for their IC_50_ values against three cancer cell lines (A549, PC-3 and MCF-7). Most of the target compounds exhibited moderate cytotoxicity against the three cancer cell lines. Two selected compounds **14b**, **14c** were further tested for their activity against PI3Kα kinase, and the results indicated that compound **14c** showed inhibitory activity against PI3Kα kinase with an IC_50_ value of 1.25 μM. Structure-activity relationships (SARs) and pharmacological results indicated that the replacement of the thiopyranopyrimidine with an imidazopyrazine was beneficial for the activity and the position of aryl group has a significant influence to the activity of these compounds. The compounds **13a**–**g** in which an aryl group substituted at the C-4 position of the pyridine ring were more active than **12a**–**g** substituted at the C-5 position. Moreover, the cytotoxicity of compounds **14a**–**g** bearing phenylpyrimidine-carboxamides was better than that of the compounds **12a**–**g**, **13a**–**g** bearing phenylpyridine-carboxamides. Furthermore, the substituents on the benzene ring also had a significant impact on the cytotoxicity and the pharmacological results showed that electron donating groups were beneficial to the cytotoxicity.

## 1. Introduction

**Figure 1:**
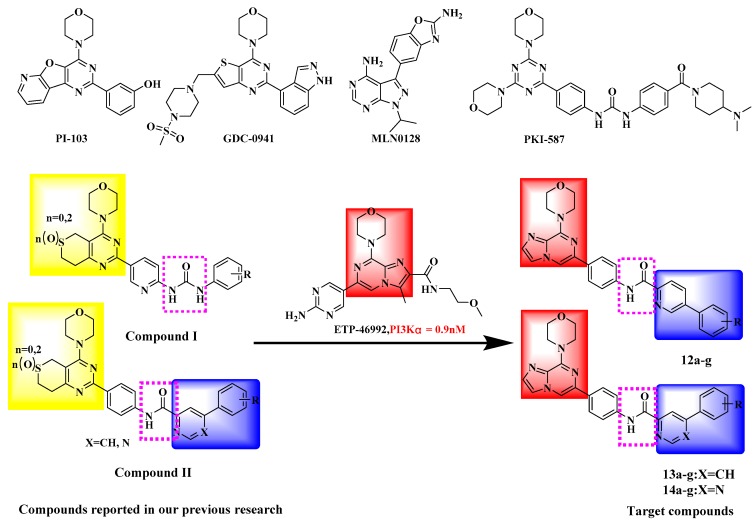
Structures of some reported PI3K pathway inhibitors and design strategy of the target compounds.

At present, with the in-depth study of cancer pathogenesis at the molecular level, molecular targeted therapy has become a research hotspot. Numerous molecular signaling pathways have been shown to be associated with the development and progression of cancer. The PI3K-Akt-mTOR signaling pathway is one of them, which was found frequently activated in different cancers. It can promote the development of tumor by inhibiting cell apoptosis, promoting angiogenesis, cell proliferation and invasion [1,2]. Therefore, the inhibition of the PI3K-Akt-mTOR pathway can effectively suppress the occurrence and development of cancer. Currently, a number of inhibitors acting on this pathway have been reported, such as PI-103, GDC-0941, MLN0128, PKI-587 and ETP46992 [3,4,5,6,7] (Figure 1). Among them, PI-103 was the first PI3K/mTOR dual inhibitor with IC_50_ values of 8 and 20 nM against PI3Kα and mTOR, respectively [3]. GDC-0941 was the first PI3K inhibitor entering clinical studies and was developed by Genentech based on the modification and optimization of PI-103 [4].

In our previous research [8,9,10,11], several series of thiopyranopyrimidine derivatives were designed and synthesized as PI3K-Akt-mTOR pathway inhibitors, such as compound **I** and compound **II**. In order to screen for compounds with excellent anti-tumor activity, further studies on these compounds were carried out in this research. According to the reported PI3K pathway inhibitors, we found that most of them contained (fused-)pyrimidine/pyrazine skeleton structure and the SARs results indicated that this skeleton was beneficial to the activity. Therefore, we replaced the thiopyranopyrimidine with an imidazopyrazine inspired by ETP46992 which is a PI3Kα inhibitor with an IC_50_ value of 0.9 nM. In addition, we maintained the amide scaffold based on the SARs results of our previous research. Then, we changed the position of aryl group on the pyridine/pyrimidine to investigate the effect on activity. Lastly, we introduced various substituents on the benzene ring to investigate their effect on anti-tumor activity. As a result, three series of 8-morpholinoimidazo[1,2-*a*]pyrazine derivatives bearing phenylpyridine-carboxamides or phenylpyrimidine-carboxamides were designed and synthesized. The design strategy for all target compounds is shown in Figure 1.

Herein, we report the design, synthesis and the cytotoxicity of all target compounds against three human cancer cell lines A549 (lung cancer), PC-3 (prostate cancer), and MCF-7 (breast cancer), as well as the activity of two selected compounds **14b**, **14c** against PI3Kα kinase. In addition, a molecular modeling docking study of the most promising compound **14c** with PI3Kα kinase was performed.

## 2. Results and Discussion

**Scheme 1:**
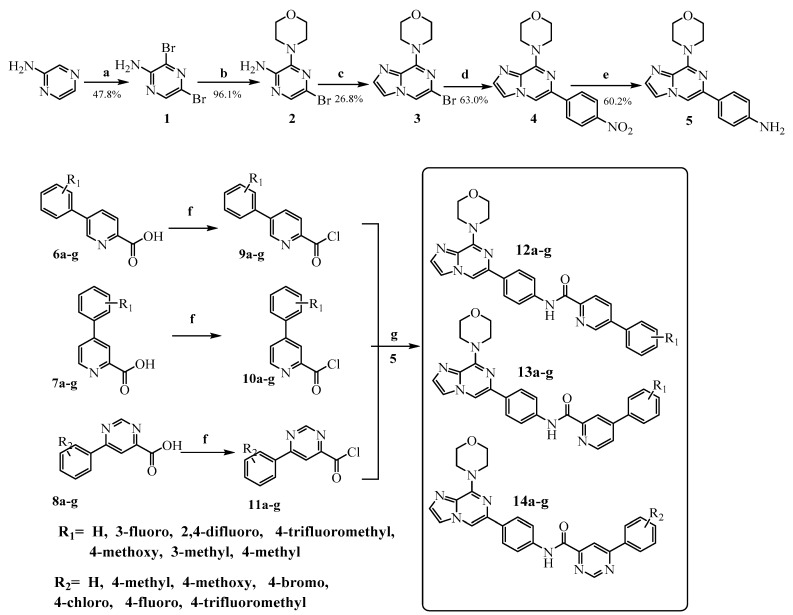
Synthetic routes of target compounds **12a**–**g**, **13a**–**g** and **14a**–**g**. *Reagents and conditions*: (a) Br_2_, pyridine, CH_2_Cl_2,_ 40 °C, 1.5 h; (b) morpholine, 80 °C, reflux, 1 h; (c) chloroacetaldehyde, isopropanol, 45 °C~65 °C, 2 h; (d) (1) bis(pinacolato)diboron, KAc, Pd(PPh_3_)Cl_2_, *p*-bromonitrobenzene, 1,2-dimethoxyethane, reflux, 2–3 h; (2) H_2_O, Na_2_CO_3_, Pd(PPh_3_)_2_Cl_2_, reflux, 6–8 h; (e) hydrazine hydrate, FeCl_3_, Activated carbon, ethanol, reflux, 1–3 h; (f) thionyl chloride, reflux, 1 h; (g) DIPEA, CH_2_Cl_2_, 0.5 h.

The preparations of target compounds **12a**–**g**, **13a**–**g** and **14a**–**g** were described in Scheme 1. The key intermediate **5** was synthesized from 2-aminopyrazine in five steps. The 2-aminopyrazine was brominated to yield 3,5-dibromopyrazin-2-amine (**1**) and the 3-bromine atom was then substituted with morpholine to give compound **2**. Subsequently, compound **2** was cyclized with chloroacetaldehyde to give compound **3**. Compound **3** was coupled with *p*-bromonitrobenzene by Suzuki coupling and then reduced with hydrazine hydrate to obtain the key intermediate compound **5**. The 5-(substituted phenyl)picolinic acids **6a**–**g**, 4-(substituted phenyl)picolinic acids **7a**–**g** and 6-(substituted phenyl)pyrimidine-4-carboxylic acids **8a**–**g** were synthesized through the procedures reported in our previous research [11,12]. Carboxylic acids **6a**–**g**, **7a**–**g** and **8a**–**g** were converted into the corresponding acyl chlorides and then immediately amidated with compound **5** to obtain the target compounds **12a**–**g**, **13a**–**g** and **14a**–**g**, respectively (Scheme 1). The yields for each target compound are listed in Table 1.

### 2.1. Biological Evaluation

The target compounds **12a**–**g**, **13a**–**g** and **14a**–**g** were evaluated for their in vitro cytotoxicity against three cancer cell lines (A549, PC-3 and MCF-7) by the standard MTT assay, with compound **II** and GDC-0941 as positive controls. Two selected compounds (**14b**, **14c**) were further tested for PI3Kα kinase inhibitory activity together with reference compound **II**, GDC-0941 and PI-103 by a Kinase-Glo^®^ Luminescent Kinase Assay. The results expressed as inhibition rates or IC_50_ values are summarized in Table 1 and Table 2, where the values are the average of at least two independent experiments. 

From Table 1, we can see that most of the target compounds showed moderate cytotoxicity against the three cancer cell lines, with IC_50_ values ranging from 6.39 to 74.9 μM and most of them showed more activity against the A549 cancer cell line than the other two cancer cell lines (PC-3, MCF-7). The IC_50_ values of the most promising compound **14c** against the A549 cancer cell lines was 6.39 μM, which was superior to the previously prepared compound **II** (IC_50_ values 8.37 μM). This means that the replacement of the thiopyranopyrimidine with an imidazopyrazine was beneficial to the cytotoxicity. Comparing the activity of the three series of target compounds, we found that an aryl group at the pyridine C-4 position (compounds **13a**–**g**) was more active than that at the C-5 position (compounds **12a**–**g**). Besides, the data of Table 1 show that the cytotoxicity of compounds **14a**–**g** bearing a phenylpyrimidine-carboxamides was better than that of compounds **12a**–**g**, **13a**–**g** bearing a phenylpyridine-carboxamide. In addition, due to the different substituents on the benzene ring, the cytotoxicity of the same class of compounds varied too. The results showed that the compounds containing electron donating groups (i.e., **12e**–**g**, **13e**–**g** and **14a**–**c**) exhibited higher cytotoxicity than the compounds containing electron-withdrawing ones (i.e., **12b**–**d**, **13b**–**d** and **14d**–**g**).

The activity of the selected compounds **14b**, **14c** as well as the positive control compounds against the PI3Kα kinase is shown in Table 2. The activity of compound **14b** against PI3Kα kinase was moderate, with an inhibitory value of 58.0% at 10 μM. The most promising compound **14c** showed an IC_50_ value of 1.25 μM against PI3Kα kinase, which was 6-fold more active than the lead compound **II** (IC_50_ values 7.39 μM). This indicated that the replacement of the thiopyranopyrimidine structure with the imidazopyrazine moiety enhanced the activity against PI3Kα kinase.

As described above, the replacement of the thiopyranopyrimidine with an imidazopyrazine was beneficial to the activity of these derivatives. In addition, the series of compounds bearing phenylpyrimidine-carboxamide structures showed the best activity among all three series of target compounds. Moreover, electron donating substituents on the benzene ring were beneficial to the cytotoxicity. Comparing Table 1 and Table 2, we found that the differences in cytotoxicity between compounds **14b** and **14c** were not particularly significant, but they were significant for PI3Kα activity. This suggested that compound **14b** may have potential activity on other targets, which may be an interesting direction in our future studies.

### 2.2. Molecular Docking Study

To explore the binding modes of the target compounds with the active site of PI3Kα, molecular docking simulation studies were carried out by using the SURFLEX-DOCK module of the SYBYL software package. Based on the results of the in vitro activity tests, we selected compound **14c** as a ligand example, and the structure of PI3Kα (PDB ID code: 4L23) was selected as the docking model [9,10].

**Figure 2:**
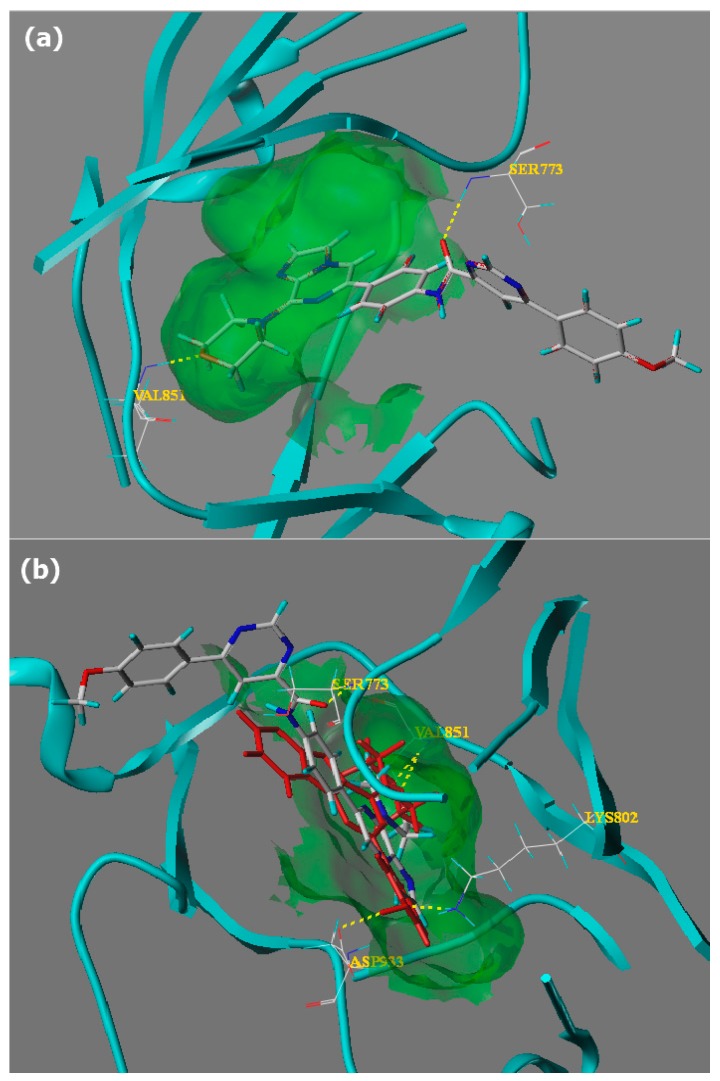
Binding models: The proteins were displayed by cyan ribbon. Hydrogen bonds were showed in dashed lines (yellow). (**a**) Binding models of compound **14c** (shown in Capped Sticks) with PI3Kα; (**b**) Binding models of compound **14c** and native ligand PI 103 (shown in red Sticks) with PI3Kα.

The binding mode of compound **14c** with PI3Kα is shown in Figure 2a,b. As depicted in Figure 2a, the imidazopyrazine scaffold and morpholine group of compound **14c** were tightly packed into the channel formed by the amino acid residues of PI3Kα. We found that the compound **14c** formed two hydrogen bonds with residues of the PI3Kα molecule: the oxygen atom of morpholine with the Val851 residue and the carbonyl of the amide scaffold with the SER773 residue. 

This result indicates that the morpholine and the amide scaffold are very important to maintain the activity against PI3Kα kinase of the target compounds. In addition, according to the comparison of docking results of the compound **14c** and PI-103 (Figure 2b), we see that the main skeleton structures of compound **14c** and the PI-103 molecule scaffold were almost completely overlapped, especially the morpholine group. This result suggested that the replacement of the thiopyranopyrimidine with an imidazopyrazine was advantageous in improving the activity. What’s more, the docking results also suggest that the benzene ring of phenylpyridine/phenylpyrimidine cannot bind well to PI3Kα, which may be one reason why the anti-tumor activity of these compounds is not particularly good. For this reason, we may be able to improve the anti-tumor activity of them by modify this part in the future work.

## 3. Experimental Section

### 3.1. General Information

All melting points were obtained on a Büchi Melting Point B-540 apparatus (Büchi Labortechnik, Flawil, Switzerland). NMR spectra were recorded using a Bruker 400 MHz spectrometer (Bruker Bioscience, Billerica, MA, USA) with TMS as an internal standard. Mass spectra (MS) were taken in ESI mode on Agilent 1100 LC–MS (Agilent, Palo Alto, CA, USA). TLC analysis was carried out on silica gel plates GF254 (Qingdao Haiyang Chemical, Qingdao, China). All the reagents were obtained from commercial suppliers and used without purification, unless otherwise specified. 

### 3.2. Chemistry

*3,5-Dibromo-2-aminopyrazine* (**1**)

A solution of dichloromethane (200 mL) and pyridine (25.3 mL, 0.315 mol) was added to a three-necked flask containing 2-aminopyrazine (14.27 g, 0.15 mol) and stirred well. In the dark and while refluxing a solution of bromine (16.2 mL, 0.315 mol) in dichloromethane (100 mL) was slowly added dropwise to the three-necked flask. About 1 h later the addition finished and the mixture was refluxed at 40 °C for 30 min more. After TLC monitoring indicated the reaction was complete, the reaction mixture was cooled to room temperature and distilled water (50 mL) was added and the mixture was stirred vigorously for 10 min. Then the organic layer was collected and washed twice with distilled water. Silica gel (10 g) and activated carbon (1 g) were added to the organic layer and the mixture was decolorized under reflux for 30 min. After hot filtration, the filtrate was collected and vacuum distilled. The residue was refluxed with *n*-hexane (45 mL) for 2 h, filtered while hot again and the solid product was dried and weighed to give 18.15 g of a pale yellow solid (47.8% yield). ^1^H-NMR (DMSO-*d*_6_) δ 8.14 (s, 1H), 7.01 (s, 2H).

*3-Morpholino-5-bromo-2-aminopyrazine* (**2**)

A mixture of morpholine (50 mL) and 3,5-dibromo-2-aminopyrazine (12.50 g) was refluxed for 1 h until the reaction was complete by TLC analysis. The solution was cooled to room temperature and added to a beaker containing ice water (300 mL) with continuous stirring. The solid was precipitated and filtered. After drying, 12.30 g of a yellow solid with a metallic luster was obtained (a yield of 96.1%). ^1^H-NMR (DMSO-*d*_6_) δ 7.70 (s, 1H), 6.28 (s, 2H), 3.85–3.62 (m, 4H), 3.04 (d, *J* = 4.0 Hz, 4H).

*4-(6-Bromoimidazo[1,2-a]pyrazin-8-yl)morpholine* (**3**)

Chloroacetaldehyde (4.4 g) was mixed with isopropanol (15 mL) and a portion of the mixture (10 mL) was added to a round bottom flask containing compound **2** (1.7 g). Then the temperature was raised to 45 °C and stirring was continued. After 1 h, the remaining solution was added and the temperature raised to 65 °C with continuous stirring. After TLC indicated the reaction was finished, the reaction was stopped and cooled to room temperature. Then the solution was added into a beaker containing ice water (300 mL) with continuous stirring. The brown-yellow solid that precipitated was filtered and dried to give the product in a yield of 26.8%. ^1^H-NMR (DMSO-*d*_6_) δ 7.93 (s, 1H), 7.25 (d, *J* = 7.3 Hz, 1H), 7.01 (d, *J* = 7.3 Hz, 1H), 3.45 (s, 4H), 3.77 (d, *J* = 4.3 Hz, 4H).

*4-(6-(4-Nitrophenyl)imidazo[1,2-a]pyrazin-8-yl)morpholine* (**4**)

Compound **4** was prepared using the synthetic method described in our previous article [11]. Briefly, a solution of 1-bromo-4-nitrobenzene (2 g, 0.01 mol), bis(pinacolato) diboron (3.8 g, 0.015 mol), potassiumacetate (2.9 g, 0.03 mol) and bistriphenylphosphine palladium dichloride (0.18 g, 0.25 mmol) was added to 1,4-dioxane (50 mL) and heated to 80 °C. The reaction was continued for 3 h under N_2_ and monitored by TLC. H_2_O (20 mL) was added to the solution and continued for 5 min. Subsequently, compound **3** (1.7 g, 0.006 mol), Na_2_CO_3_ (2.1 g, 0.02 mol) and bistriphenylphosphine dichloride (0.18 g, 0.25 mmol) were added successively, then the mixture was heated to 100 °C. The reaction was continued for about 8 h. The mixture was cooled and concentrated under vacuum. H_2_O (100 mL) was added, stirred for 30 min and then filtered. The filter cake was decolorized with activated carbon (5 g) and silica gel (2 g) in a mixed solvent (CH_2_Cl_2_:CH_3_OH = 5:1, 100 mL), filtered while hot and concentrated under vacuum to obtain **4** with a yield of 63.0%. ESI-MS *m*/*z*: [M + H]^+^ 326.1. ^1^H-NMR (DMSO-*d*_6_) δ 8.78 (d, *J* = 8.2 Hz 2H), 8.33 (d, *J* = 8.2 Hz 2H), 8.01 (s, 1H), 7.20 (d, *J* = 8.7 Hz, 1H), 6.95 (d, *J* = 8.7 Hz, 1H), 3.91 (s, 4H), 3.77 (s, 4H).

*4-(8-Morpholinoimidazo[1,2-a]pyridin-6-yl)aniline* (**5**)

The synthesis of compound **5** is similar to the synthetic method reported in our previous article [11]. Compound **4** (3.3 g, 0.01 mol) was refluxed with hydrazine hydrate (5 g, 0.1 mol), ferric chloride (0.5 g, 0.002 mol) and an appropriate amount of activated carbon in ethanol (50 mL) for 30 min–1 h and monitored by TLC. After filtering while hot and concentrating under vacuum compound **5** was obtain in a yield of 60.2%. ESI-MS *m*/*z*: [M + H]^+^ 296.2. [M + H]^+^ 326.1. ^1^H-NMR (DMSO-*d*_6_) δ 8.12 (d, *J* = 8.5 Hz 2H), 7.89 (s, 1H), 7.12 (d, *J* = 8.3 Hz, 1H), 6.99 (d, *J* = 8.3 Hz, 1H), 6.60 (d, *J* =8.5 Hz 2H), 5.56 (s, 2H), 3.75 (d, *J* = 3.5 Hz, 4H), 3.32 (s, 4H).

*General Procedure for the Preparation of Compounds* **6a**–**g**, **7a**–**g**, **8a**–**g**, **9a**–**g**, **10a**–**g** *and* **11a**–**g**

Compounds **6a**–**g**, **7a**–**g** and **8a**–**g** were synthesized according to the procedures reported by our research group [11,12]. Then compounds **6a**–**g**, **7a**–**g** and **8a**–**g** (0.02 mmol) were dissolved in thionyl chloride (8 mL) and refluxed for 1 h. The reaction mixture was evaporated to yield the corresponding chloride which was dissolved in dichloromethane (10 mL). The solution of **9a**–**g**, **10a**–**g** or **11a**–**g** was used for the next step without further purification.

*General Procedure for the Preparation of the Target Compounds* **12a**–**g**, **13a**–**g** *and* **14a**–**g**

A solution of phenylpyrimidine chloride **9a**–**g**, **10a**–**g** or **11a**–**g** in dichloromethane (10 mL) was added dropwise to a solution of **5** (0.12 mol) and *N*,*N*-diisopropylethylamine (0.048 mol) in dichloromethane (10 mL) in an ice bath. Upon completion of the addition, the reaction mixture was removed from the ice bath, held at room temperature for 15 min and monitored by thin-layer chromatography (TLC). The mixture was washed with 10% K_2_CO_3_ (50 mL × 3) followed by brine (50 mL × 1), and the organic layer was separated, dried over anhydrous sodium sulfate, and evaporated to yield the target compounds **12a**–**g**, **13a**–**g** and **14a**–**g** which were recrystallized from isopropanol. In addition, all the target compounds were purified by silica gel column chromatography (CH_2_Cl_2_:CH_3_OH = 100:1).

*N-(4-Morpholinoimidazo[1,2-a]pyridin-6-yl) phenyl)-4-phenylpyridine amide* (**12a**). A yellow solid; yield: 96.1%; m.p. 268–270 °C; ESI-MS [M + H]^+^ *m*/*z*: 477.2; ^1^H-NMR (CDCl_3_) δ 10.46 (s, 1H), 9.06 (s, 1H), 8.86 (s, 2H), 8.39 (d, *J* = 8.0 Hz, 1H), 8.28 (d, *J* = 8.1 Hz, 1H), 8.22 (d, *J* = 8.2 Hz, 1H), 8.13–8.04 (m, 4H), 7.96 (s, 3H), 7.83 (s, 2H), 4.21 (s, 4H), 4.01 (s, 4H).

*5-(3-Fluorophenyl)-N-(4-(8-morpholinoimidazo[1,2-a]pyrazin-6-yl)phenyl)picolinamide* (**12b**). A yellow solid; yield: 79.4%; m.p. 261–264 °C; ESI-MS [M + H]^+^ *m*/*z*: 495.2; ^1^H-NMR (400 MHz, CDCl_3_) δ 10.18 (s, 1H), 8.85 (s, 2H), 8.40 (d, *J* = 8.1 Hz, 2H), 8.29 (d, *J* = 8.1 Hz, 1H), 8.09 (s, 3H), 7.88 (s, 1H), 7.75 (s, 1H), 7.52 (d, *J* = 5.1 Hz, 1H), 7.45 (s, 1H), 7.36 (d, *J* = 9.2 Hz, 1H), 7.20 (d, *J* = 9.0 Hz, 1H), 4.17 (s, 4H), 4.03 (s, 4H).

*5-(2,4-Difluorophenyl)-N-(4-(8-morpholinoimidazo[1,2-a]pyrazin-6-yl)phenyl)picolinamide* (**12c**). A pale yellow solid; yield: 90.2%; m.p. 255–257 °C; ESI-MS [M + H]^+^ *m*/*z*: 513.2; ^1^H-NMR (DMSO-*d*_6_) δ 10.97–10.71 (m, 1H), 8.86 (s, 1H), 8.35 (d, *J* = 9.2 Hz, 1H), 8.22 (s, 1H), 8.04 (s, 1H), 7.84–7.71 (m, 1H), 7.60 (d, *J* = 12.2 Hz, 2H), 7.51–7.37 (m, 1H), 7.32–7.17 (m, 1H), 6.51 (s, 2H), 6.30 (s, 2H), 3.92 (s, 4H), 3.74 (s, 4H).

*N-(4-(8-Morpholinoimidazo[1,2-a]pyrazin-6-yl)phenyl)-5-(4-(trifluoromethyl)phenyl)picolinamide* (**12d**). A gray solid; yield: 83.5%; m.p. 233–235 °C; ESI-MS [M + H]^+^ *m*/*z*: 545.2; ^1^H-NMR (CDCl_3_) δ 10.46 (s, 1H), 8.88 (s, 2H), 8.33 (d, *J* = 8.0 Hz, 2H), 8.23 (d, *J* = 14.9 Hz, 1H), 8.12 (s, 2H), 7.99 (d, *J* = 22.6 Hz, 5H), 7.56 (s, 1H), 7.48 (s, 1H), 4.08 (d, *J* = 18.4 Hz, 8H).^13^C-NMR (DMSO-*d*_6_) δ 162.66, 157.43, 149.99, 147.87, 147.28, 138.75, 137.36, 136.99, 136.53, 135.92, 132.97, 132.96, 132.52, 132.29, 128.69 (2C), 128.68 (2C), 128.52, 126.28 (2C), 123.14, 120.76 (2C), 116.11, 66.73 (2C),46.71 (2C).

*5-(4-Methoxyphenyl)-N-(4-(8-morpholinoimidazo[1,2-a]pyrazin-6-yl)phenyl)picolinamide* (**12e**). A pale yellow solid; yield: 93.3%; m.p. 255–257 °C; ESI-MS [M + H]^+^ *m*/*z*: 507.2; ^1^H-NMR (DMSO-*d*_6_) δ 11.05 (s, 1H), 8.74 (d, *J* = 5.1 Hz, 1H), 8.57 (s, 1H), 8.47 (s, 1H), 8.03 (d, *J* = 8.6 Hz, 2H), 8.02 (s, 2H), 7.87 (d, *J* = 9.8 Hz, 2H), 7.86 (d, *J* = 8.6 Hz, 2H), 7.51 (s, 1H), 7.14 (d, *J* = 8.6 Hz, 2H), 4.32 (s, 4H), 4.19 (s, 2H), 3.85 (d, *J* = 3.9 Hz, 3H).

*N-(4-(8-Morpholinoimidazo[1,2-a]pyrazin-6-yl)phenyl)-5-(m-tolyl)picolinamide* (**12f**). A pale yellow solid; yield: 72.6%; m.p. 241–243 °C; ESI-MS [M + H]^+^ *m*/*z*: 491.2; ^1^H-NMR (CDCl_3_) δ 10.17 (s, 1H), 8.84 (s, 1H), 8.36 (d, *J* = 8.0 Hz, 1H), 8.30–8.22 (m, 1H), 8.20 (d, *J* = 7.5 Hz, 1H), 8.10 (d, *J* = 9.8 Hz, 2H), 8.06 (s, 2H), 7.93 (d, *J* = 8.2 Hz, 2H), 7.74 (s, 1H), 7.56 (d, *J* = 7.3 Hz, 3H), 4.25 (s, 4H), 3.98 (s, 4H), 2.44 (s, 3H).

*N-(4-(8-Morpholinoimidazo[1,2-a]pyrazin-6-yl)phenyl)-5-(p-tolyl)picolinamide* (**12g**). A pale yellow solid; yield: 89.1%; m.p. 258–260 °C; ESI-MS [M + H]^+^ *m*/*z*: 491.2; ^1^H-NMR (DMSO-*d*_6_) δ 10.79 (s, 1H), 9.02 (d, *J* = 9.4 Hz, 2H), 8.59 (s, 1H), 8.35 (d, *J* = 8.1 Hz, 1H), 8.24 (d, *J* = 8.1 Hz, 1H), 8.05 (d, *J* = 8.7 Hz, 2H), 7.77 (s, 2H), 7.59 (s, 2H), 7.37 (t, *J* = 8.5 Hz, 3H), 4.30 (s, 4H), 3.80 (s, 4H), 2.40 (s, 3H).

*N-(4-(8-Morpholinoimidazo[1,2-a]pyrazin-6-yl)phenyl)-4-phenylpicolinamide* (**13a**). A yellow solid; yield: 97.0%; m.p. 261–262 °C; ESI-MS [M + H]^+^ *m*/*z*: 477.2; ^1^H-NMR (DMSO-*d*_6_) δ 9.55 (s, 1H), 7.59 (d, *J* = 5.0 Hz, 1H), 7.34 (s, 1H), 7.20 (s, 1H), 6.79 (d, *J* = 7.8 Hz, 5H), 6.74–6.65 (m, 3H), 6.34 (dd, *J* = 8.4, 4.3 Hz, 4H), 3.07 (s, 4H), 2.61–2.51 (m, 4H).

*4-(3-Fluorophenyl)-N-(4-(8-morpholinoimidazo[1,2-a]pyrazin-6-yl)phenyl)picolinamide* (**13b**). A white solid; yield: 88.0%; m.p. 237–239 °C; ESI-MS [M + H]^+^ *m*/*z*: 495.2; ^1^H-NMR (DMSO-*d*_6_) δ 10.85 (s, 1H), 8.84 (d, *J* = 5.0 Hz, 1H), 8.80 (d, *J* = 5.0 Hz, 1H), 8.60 (s, 1H), 8.45 (s, 1H), 8.33 (s, 1H), 8.06 (dd, *J* = 5.7, 3.5 Hz, 3H), 7.96 (s, 1H), 7.85–7.79 (m, 2H), 7.63 (d, *J* = 7.7 Hz, 1H), 7.45–7.33 (m, 2H), 4.30 (s, 4H), 3.80 (d, *J* = 4.2 Hz, 4H).

*4-(2,4-Difluorophenyl)-N-(4-(8-morpholinoimidazo[1,2-a]pyrazin-6-yl)phenyl)picolinamide* (**13c**). A pale yellow solid; yield: 91.2%; m.p. 266–267 °C; ESI-MS [M + H]^+^ *m*/*z*: 513.2; ^1^H-NMR (DMSO-*d*_6_) δ 10.76 (s, 1H), 8.79 (d, *J* = 5.1 Hz, 1H), 8.52 (s, 1H), 8.26 (s, 1H), 7.96 (d, *J* = 7.3 Hz, 4H), 7.89 (s, 1H), 7.85–7.74 (m, 2H), 7.52 (s, 1H), 7.43 (d, *J* = 9.6 Hz, 1H), 7.26 (t, *J* = 8.3 Hz, 1H), 4.23 (s, 4H), 3.74 (d, *J* = 4.3 Hz, 4H).

*N-(4-(8-Morpholinoimidazo[1,2-a]pyrazin-6-yl)phenyl)-4-(4-(trifluoromethyl)phenyl)picolinamide* (**13d**). A pale yellow solid; yield: 81.8%; m.p. 269–272 °C; ESI-MS [M + H]^+^ *m*/*z*: 545.2; ^1^H-NMR (DMSO-*d*_6_) δ 10.86 (s, 1H), 8.88 (d, *J* = 5.0 Hz, 1H), 8.59 (s, 1H), 8.49 (s, 1H), 8.15 (d, *J* = 8.0 Hz, 2H), 8.13–8.07 (m, 2H), 8.03 (d, *J* = 11.5 Hz, 3H), 8.00 (s, 1H), 7.95 (d, *J* = 8.4 Hz, 2H), 7.59 (s, 1H), 4.30 (s, 4H), 3.80 (d, *J* = 4.0 Hz, 4H).

*4-(4-Methoxyphenyl)-N-(4-(8-morpholinoimidazo[1,2-a]pyrazin-6-yl)phenyl)picolinamide* (**13e**). A pale yellow solid; yield: 93.1%; m.p. 257–260 °C; ESI-MS [M + H]^+^ *m*/*z*: 507.2; ^1^H-NMR (DMSO-*d*_6_) δ 10.82 (s, 1H), 8.76 (d, *J* = 5.1 Hz, 1H), 8.60 (s, 1H), 8.40 (s, 1H), 8.05 (d, *J* = 8.6 Hz, 2H), 8.02 (s, 2H), 7.97 (d, *J* = 9.8 Hz, 2H), 7.91 (d, *J* = 8.6 Hz, 2H), 7.59 (s, 1H), 7.14 (d, *J* = 8.6 Hz, 2H), 4.30 (s, 4H), 3.85 (s, 2H), 3.80 (d, *J* = 3.9 Hz, 3H).

*N-(4-(8-Morpholinoimidazo[1,2-a]pyrazin-6-yl)phenyl)-4-(m-tolyl)picolinamide* (**13f**). A pale red solid; yield: 91.5%; m.p. 233–234 °C; ESI-MS [M + H]^+^ *m*/*z*: 491.2; ^1^H-NMR (DMSO-*d*_6_) δ 10.82 (s, 1H), 8.79 (d, *J* = 4.9 Hz, 1H), 8.59 (s, 1H), 8.42 (s, 1H), 8.30 (s, 1H), 8.05 (d, *J* = 8.4 Hz, 3H), 7.83 (d, *J* = 8.1 Hz, 3H), 7.59 (s, 1H), 7.43–7.36 (m, 3H), 4.30 (s, 4H), 3.80 (d, *J* = 3.7 Hz, 4H), 2.40 (s, 3H).

*N-(4-(8-Morpholinoimidazo[1,2-a]pyrazin-6-yl)phenyl)-4-(p-tolyl)picolinamide* (**13g**). A brown solid; yield: 98.0%; m.p. 271–273 °C; ESI-MS [M + H]+ *m*/*z*: 491.2; ^1^H-NMR (DMSO-*d*_6_) δ 9.87 (s, 1H), 7.90 (d, *J* = 5.2 Hz, 1H), 7.56 (s, 1H), 7.44 (s, 1H), 7.22–7.15 (m, 3H), 6.95 (d, *J* = 8.5 Hz, 3H), 6.73 (s, 1H), 6.54 (s, 4H), 3.45 (s, 4H), 3.15–2.68 (m, 4H), 2.33 (s, 3H).^13^C-NMR (DMSO-*d*_6_) δ 162.98, 151.15, 149.90, 149.64, 149.31, 147.87, 140.08, 138.78, 135.91, 134.03, 132.94, 132.30, 130.48 (2C), 127.35 (2C), 127.31, 126.29 (2C), 120.70, 119.64 (2C), 116.13, 116.11, 66.73 (2C), 46.70 (2C), 21.29.

*N-(4-(8-Morpholinoimidazo[1,2-a]pyrazin-6-yl)phenyl)-6-phenylpyrimidine-4-carboxamide* (**14a**). A pale yellow solid; yield: 82.7%; m.p. 272–275 °C; ESI-MS [M + H]^+^ *m*/*z*: 478.1; ^1^H-NMR (DMSO-*d*_6_) δ 10.79 (s, 1H), 8.75 (d, *J* = 5.1 Hz, 1H), 8.58 (s, 1H), 8.39 (s, 1H), 8.04 (d, *J* = 8.7 Hz, 2H), 8.01 (s, 1H), 7.98 (d, *J* = 6.3 Hz, 2H), 7.95 (s, 1H), 7.89 (d, *J* = 8.5 Hz, 2H), 7.58 (s, 1H), 7.13 (d, *J* = 8.5 Hz, 2H), 4.29 (s, 4H), 3.80 (d, *J* = 4.3 Hz, 4H).

*N-(4-(8-Morpholinoimidazo[1,2-a]pyrazin-6-yl)phenyl)-6-(p-tolyl)pyrimidine-4-carboxamide* (**14b**). A pale yellow solid; yield: 79.5%; m.p. 222–225 °C; ESI-MS [M + H]^+^ *m*/*z*: 492.2; ^1^H-NMR (DMSO-*d*_6_) δ 10.82 (s, 1H), 8.77 (dd, *J* = 22.5, 5.1 Hz, 2H), 8.58 (s, 1H), 8.41 (s, 1H), 8.27 (s, 1H), 7.94–7.87 (m, 2H), 7.58 (s, 2H), 7.41 (t, *J* = 8.3 Hz, 4H), 4.29 (s, 4H), 3.79 (s, 4H), 1.25 (d, *J* = 6.5 Hz, 3H).

*6-(4-Methoxyphenyl)-N-(4-(8-morpholinoimidazo[1,2-a]pyrazin-6-yl)phenyl)pyrimidine-4-carboxamide* (**14c**). A pale brown solid; yield: 77.8%; m.p. 269–271 °C; ESI-MS [M + H]^+^ *m*/*z*: 508.2; ^1^H-NMR (DMSO-*d*_6_) δ 10.96 (s, 1H), 9.38 (s, 1H), 8.57 (s, 1H), 8.32 (d, *J* = 8.0 Hz, 2H), 8.03 (dd, *J* = 14.7, 8.4 Hz, 4H), 7.96 (s, 1H), 7.08 (d, *J* = 1.7 Hz, 2H), 7.05 (d, *J* = 1.6 Hz, 2H), 4.29 (s, 4H), 3.87 (s, 3H), 3.80 (s, 4H). ^13^C-NMR (DMSO-*d*_6_) δ 162.80, 162.53, 162.06, 158.73, 158.48, 157.98, 157.00, 138.37, 133.36, 132.96, 132.29, 129.59 (2C), 128.25, 128.20, 126.30 (2C), 120.98 (2C), 116.12, 114.98 (3C), 66.73 (2C), 55.95, 46.70 (2C).

*6-(4-Bromophenyl)-N-(4-(8-morpholinoimidazo[1,2-a]pyrazin-6-yl)phenyl)pyrimidine-4-carboxamide* (**14d**). A white solid; yield: 90.9%; m.p. 221–223 °C; ESI-MS [M + H]^+^ *m*/*z*: 557.1; ^1^H-NMR (DMSO-*d*_6_) δ 10.91 (s, 1H), 9.49 (s, 1H), 8.65 (s, 1H), 8.59 (d, *J* = 11.9 Hz, 2H), 8.38 (d, *J* = 8.3 Hz, 2H), 8.00–7.94 (m, 3H), 7.85 (d, *J* = 8.3 Hz, 2H), 7.59 (d, *J* = 2.7 Hz, 2H), 4.30 (s, 4H), 3.79 (d, *J* = 4.0 Hz, 4H).

*6-(4-Chlorophenyl)-N-(4-(8-morpholinoimidazo[1,2-a]pyrazin-6-yl)phenyl)pyrimidine-4-carboxamide* (**14e**). A pale yellow solid; yield: 93.2%; m.p. 235–237 °C; ESI-MS [M + H]^+^ *m*/*z*: 513. 1; ^1^H-NMR (DMSO-*d*_6_) δ 10.81 (s, 1H), 8.78 (d, *J* = 5.1 Hz, 1H), 8.58 (s, 1H), 8.41 (s, 1H), 8.05 (d, *J* = 8.8 Hz, 2H), 8.00 (d, *J* = 7.5 Hz, 2H), 7.95 (s, 1H), 7.83 (d, *J* = 8.0 Hz, 2H), 7.58 (s, 1H), 7.41 (d, *J* = 8.0 Hz, 2H), 4.29 (s, 4H), 3.88–3.67 (m, 4H).

*6-(4-Fluorophenyl)-N-(4-(8-morpholinoimidazo[1,2-a]pyrazin-6-yl)phenyl)pyrimidine-4-carboxamide* (**14f**). A gray solid; yield: 80.8%; m.p. 259–260 °C; ESI-MS [M + H]^+^ *m*/*z*: 496.2; ^1^H-NMR (CDCl_3_) δ 10.05 (s, 1H), 9.33 (s, 1H), 8.97 (s, 1H), 8.63 (s, 1H), 8.17 (dd, *J* = 18.2, 8.6 Hz, 2H), 8.10–7.83 (m, 5H), 7.75 (d, *J* = 5.2 Hz, 1H), 7.55 (d, *J* = 8.0 Hz, 2H), 4.32 (s, 4H), 3.96 (s, 4H).

*N-(4-(8-Morpholinoimidazo[1,2-a]pyrazin-6-yl)phenyl)-6-(4-(trifluoromethyl)phenyl)pyrimidine-4-carboxamide* (**14g**). A white solid; yield: 77.6%; m.p. 278–281 °C; ESI-MS [M + H]^+^ *m*/*z*: 546.2; ^1^H-NMR (DMSO-*d*_6_) δ 10.86 (s, 1H), 9.86 (d, *J* = 5.0 Hz, 1H), 8.49 (s, 1H), 8.15 (d, *J* = 8.0 Hz, 2H), 8.10 (d, *J* = 4.5 Hz, 1H), 8.03 (dd, *J* = 20.1, 8.6 Hz, 4H), 7.95 (d, *J* = 8.4 Hz, 3H), 7.59 (s, 1H), 4.30 (s, 4H), 3.80 (s, 4H).

### 3.3. In Vitro Cytotoxicity Assays 

#### 3.3.1. The Selection of Cancer Cell Lines

The studies of Yue showed that the expression of the VEGF, PI3K, Akt and mTOR genes of the PI3K-Akt-mTOR signaling pathway in non-small cell lung cancer (A549) were (40 ± 59)%, (61 ± 23)%, (77 ± 32)%, (43 ± 21)% respectively [13]. The level of this pathway in breast cancer (MCF-7) was also up to 70% [14]. In prostate cancer cells (PC-3), the missing tumor suppressor gene (PTEN) resulted in a high expression of the PI3K-Akt-mTOR signaling pathway. In addition, the studies of GDC-0941 showed it have strong inhibitory activity against A549, PC-3 and MCF-7 suggesting that these cells have a high sensitivity for PI3K-Akt—mTOR signaling pathway inhibitors [4]. Therefore, we chose these cell lines as the test cell lines for our in vitro anti-tumor experiments.

#### 3.3.2. MTT Assay In Vitro

The in vitro cytotoxic activity of all the compounds **12a**–**g**, **13a**–**g** and **14a**–**g** was evaluated with the A549, PC-3 and MCF-7 cell lines by the standard MTT assay, with compounds **II** and GDC-0941 as positive controls. The cancer cell lines were cultured in minimum essential medium (MEM) supplement with 10% fetal bovine serum (FBS). Approximately 4 × 10^3^ cells, suspended in MEM medium, were plated onto each well of a 96-well plate and incubated in 5% CO_2_ at 37 °C for 24 h. The test compounds at indicated final concentrations were added to the culture medium and the cell cultures were continued for 72 h. Fresh MTT was added to each well at a terminal concentration of 5 μg/mL and incubated with cells at 37 °C for 4 h. The formazan crystals were dissolved in 100 μL of DMSO in each well, and the absorbance at 492 nm (for the MTT formazan) and 630 nm (for the reference wavelength) was measured with an ELISA reader. All of the compounds were tested three times in each of the cell lines. The results expressed as inhibition rates or IC_50_ (half-maximal inhibitory concentration) were the averages of two determinations and calculated by using the Bacus Laboratories Incorporated Slide Scanner (Bliss) software (the Bacus Laboratories Inc. Slide Scanner (BLISS) system, Lombard, IL, USA).

### 3.4. PI3K*α* Kinase Assay

The selected compounds **14b**, **14c** were tested for their activity against PI3Kα using a Kinase-Glo^®^ Luminescent Kinase Assay, with Compound **II**, GDC-0941 and PI103 as positive controls. The kinase reaction is done in 384-well black plate. Each well is loaded with 50 μL of test items (in 90% DMSO) and 5 μL reaction buffer containing 10 μg/mL PI substrate (l-α-phosphatidylinositol, Avanti Polar Lipids, (Avanti Polar Lipids, Inc., Alabaster, AL, USA), prepared in 3% octylglucoside) and the PI3Kα protein 10 nM is then added to it. The reaction is started by the addition of 5 μL of 1 μM ATP prepared in the reaction buffer and is incubated for 60 min for p110α. It is terminated by the addition of 10 μL Kinase-Glo buffer. The plates are then read in a Synergy 2 reader (BioTek Instruments, Inc., Winooski, VT, USA) for luminescence detection. All of the compounds were tested two times [9,10]. 

### 3.5. Docking Studies

For docking purposes, we prepared the receptor protein PDB ID code: 4L23 (PI3Kα). The three-dimensional structure of the PI3Kα were obtained from the RCSB Protein Data Bank [9,10]. We built a small organic molecule set (compound **14c**) and used the Gasteiger-Hückel method to optimize the molecular force field and structure. Hydrogen atoms were added to the structure allowing for appropriate ionization at physiological pH. First of all, extract ligand substructure, then remove water and excess structure, finally, add hydrogens and fix sidechain amides. The protonated state of several important residues, such as Val851, Lys802, Ser773 and Asp933, were adjusted by using SYBYL 6.9.1 (Tripos, St. Louis, MO, USA) in favor of forming reasonable hydrogen bond with the ligand. Molecular docking analysis was carried out by the SURFLEX-DOCK module of SYBYL 6.9.1 package (Tripos) to explore the binding model for the active site of PI3Kα with its ligand. All atoms located within the range of 5.0 Å from any atom of the cofactor were selected into the active site, and the corresponding amino acid residue was, therefore, involved into the active site if only one of its atoms was selected. Other default parameters were adopted in the SURFLEX-DOCK calculations. All calculations were performed on a Silicon Graphics workstation (package version 6.9.1 on silicon graphics origin300 workstation with IRIX 6.5 operating system, San Francisco, CA, USA). Lastly, docking results and the optimized molecular docking model with the receptor proteins was obtained.

## 4. Conclusions

Three new classes of 8-morpholino imidazo[1,2-*a*]pyrazine derivatives bearing phenylpyridine-carboxamides or phenylpyrimidine-carboxamides were designed, synthesized and evaluated for their cytotoxicity against three cancer cell lines and/or PI3Kα kinase. The pharmacological results indicated that most of the synthesized compounds displayed moderate cytotoxicity against the three cell lines, with IC_50_ values ranging from 6.39 to 74.9 μM. The most promising compound **14c** showed a good inhibitory activity against PI3Kα kinase with an IC_50_ value of 1.25 ± 0.13 μM, which was more potent than the lead compound **II** (IC_50_ values 7.39 μM). Among them, the series of compounds containing phenylpyrimidine-carboxamide structures exhibited the best activity. For the other two series, the compounds with the aryl unit substituted at the pyridine C-4 position were more active than those substituted at the C-5 position. Moreover, the substituents on the benzene ring also have an important impact on the cytotoxicity. The results demonstrated that electron donating groups on the benzene ring were beneficial to the cytotoxicity. The initial SARs and docking studies with the PI3Kα molecule showed that the morpholine group, imidazopyrazine and amide scaffold were beneficial for these compounds to maintain a good activity. 

## Figures and Tables

**Table 1:**
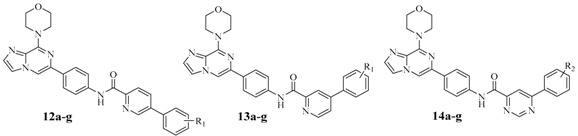
Structures and cytotoxicity of target compounds **12a**–**g**, **13a**–**g** and **14a**–**g**.

Compd.	R	Yield (%)	IC_50_ (μM) ^a^		
			A549	PC-3	MCF-7
**12a**	H	96.1	58.73 ± 1.99	50.28 ± 2.04	ND
**12b**	3-fluoro	79.4	NA	NA	NA
**12c**	2,4-difluoro	90.2	NA	NA	NA
**12d**	4-trifluoromethyl	83.5	15.27 ± 0.88	69.70 ± 1.13	ND
**12e**	4-methoxy	93.3	NA	NA	NA
**12f**	3-methyl	72.6	19.11 ± 1.10	26.78 ± 1.47	ND
**12g**	4-methyl	89.1	68.12 ± 0.89	42.32 ± 0.86	ND
**13a**	H	97.0	NA	NA	NA
**13b**	3-fluoro	88.0	NA	NA	NA
**13c**	2,4-difluoro	91.2	NA	NA	NA
**13d**	4-trifluoromethyl	81.8	41.99 ± 1.49	55.64 ± 1.32	62.48 ± 1.99
**13e**	4-methoxy	93.1	23.95 ± 0.81	59.68 ± 2.22	74.92 ± 1.14
**13f**	3-methyl	91.5	10.53 ± 0.82	65.61 ± 0.52	74.19 ± 1.86
**13g**	4-methyl	98.0	11.23 ± 1.08	NA	7.89 ± 0.81
**14a**	H	82.7	10.75 ± 1.62	NA	NA
**14b**	4-methyl	79.5	8.88 ± 0.97	25.04 ± 1.19	6.69 ± 2.04
**14c**	4-methoxy	77.8	6.39 ± 1.04	12.65 ± 0.95	10.23 ± 1.62
**14d**	4-bromo	90.9	14.36 ± 1.01	37.14 ± 0.97	NA
**14e**	4-chloro	93.2	17.38 ± 1.01	NA	NA
**14f**	4-fluoro	80.8	58.17 ± 4.67	NA	47.06
**14g**	4-trifluoromethyl	77.6	NA	96.63 ± 2.58	ND
Compound **II** ^b,c^	-	-	8.37 ± 0.10	11.34 ± 0.11	9.26 ± 0.82
GDC-0941 ^b^	-	-	6.99 ± 0.21	0.20 ± 0.08	0.07 ± 0.03

^a^ The values are an average of two separate determinations; ^b^ Used as a positive control; ^c^ Compounds reported in our previous research; NA: No Activity; ND: Not Determine.

**Table 2:** PI3Kα kinase activity of selected compounds and positive controls.

Compound No.	PI3Kα
	IC_50_ (μM) ^a^ or 10 μM Inhibitory % ^a^
**14b**	58.0 ± 4.4 ≅10 μM
**14c**	1.25 ± 0.13
Compound **II** ^b,c^	7.39 ± 0.19 [11]
GDC-0941 ^b^	0.003 [4]
PI-103 ^b^	0.075 ± 0.018

^a^ The values are an average of two separate determinations; ^b^ Used as a positive control; ^c^ Compounds reported in our previous research.
